# Protective Effect of Vitamin C against Infancy Rat Corneal Injury Caused by Acute UVB Irradiation

**DOI:** 10.1155/2020/8089273

**Published:** 2020-05-25

**Authors:** Wei Chen, Jianying Guo, Haiyi Guo, Xue Kong, Jing Bai, Pan Long

**Affiliations:** ^1^Graduate School, Fourth Military Medical University, Xi'an, Shaanxi Province 710032, China; ^2^Department of Imaging Medicine, Xijing Hospital, Fourth Military Medical University, Xi'an, Shaanxi Province 710032, China; ^3^Lourdes High School, Rochester, MN 55901, USA; ^4^Department of Ophthalmology, Xijing Hospital, Fourth Military Medical University, Xi'an, Shaanxi Province 710032, China; ^5^Department of Ophthalmology, The General Hospital of Western Theater Command, Chengdu, Sichuan Province 610083, China

## Abstract

**Purpose:**

Studies have shown that corneas of young children were more susceptible to Ultraviolet B (UVB) radiation damage. However, there exist limited information about the harm of UVB to eyes and preventive measures on infancy. Vitamin C as an antioxidant is widely used to prevent many diseases. Therefore, the aim of this study was to explore the protective effect of vitamin C on the cornea of infant rats with acute UVB injury.

**Method:**

Thirty-six infant rats were randomly divided into three groups: control (CON) group, UVB (UVB) group, and UVB+vitamin C (UVB+VitC) group. The UVB group was exposed to UVB irradiation (8 J/cm^2^, 15 min/d, 7 d) and the UVB+vitamin C group suffered the same UVB irradiation treated with vitamin C at the dose of 40 mg/kg via intraperitoneal injection. Then, corneal morphology was detected *in vivo* and *in vitro* at 7 d post-UVB exposure. Furthermore, serum inflammatory factors (IL-1, IL-6, and TNF-*α*) and oxidative status (4-HNE and MDA) were detected by ELISA, and the expression of vascular endothelial growth factor-*α* (VEGF-*α*) and superoxide dismutase (SOD) in the cornea was detected by western blot or immunofluorescent staining.

**Results:**

Slit lamp detection revealed that the area of corneal desquamation and corneal neovascularization in the UVB+VitC group was significantly less than those in the UVB group at 7 d post-UVB exposure (all *p* < 0.05). OCT results showed that the thickness of the central cornea in the UVB+VitC group was decreased than that in the UVB group (*p* < 0.05). The serum inflammatory factors (IL-1, IL-6, and TNF-*α*) and oxidative status (4-HNE and MDA) in the UVB group were significantly increased compared with the CON group (all *p* < 0.05), while those factors in the UVB+VitC group were decreased compared with those in the UVB group. Furthermore, the expression of VEGF-*α* in the UVB+VitC group was dramatically decreased compared with that in the UVB group (*p* < 0.05), and the expression of SOD2 in the UVB+VitC group was dramatically increased compared with that in the UVB group at 7 d post-UVB exposure (*p* < 0.05).

**Conclusion:**

Vitamin C could protect infant rats from corneal injury induced by UVB via alleviating corneal edema, improving corneal inflammatory reaction, and decreasing VEGF-*α* expression.

## 1. Introduction

Ultraviolet (UV) radiation is mainly classified into Ultraviolet A (UVA) radiation, Ultraviolet B (UVB) radiation, and Ultraviolet C (UVC) radiation according to the spectral wavelength. UV radiation, one part of the sunlight spectrum, is the most common cause of radiation damage to the eye, especially for corneal injury [1, 2]. A recent study demonstrated that more than 90% of UVB and 60% of UVA radiation were absorbed by the cornea [3].

The eye is one of the most sensitive parts of the human body to ultraviolet radiation. The cornea has the physiologic capability of preventing the majority of UVB radiation and protecting the lens and retina and other tissues in the eyes against UVB-induced phototoxicity and oxidative damage [4–6]. The typical UVB-induced corneal complications contain photokeratitis, damage to the epithelium, corneal stromal edema, and a number of biochemical changes, including DNA modification, protein crosslinking, enzyme inactivation, and the production of excessive reactive oxygen species (ROS) [7, 8].

A study revealed that the eyes of children were more susceptible to UV radiation damage. Epidemiological studies have shown that exposure to ultraviolet in earlier years would increase corneal diseases in adults. Evidence showed that infants living in low latitudes were more susceptible to pterygia in adulthood. Furthermore, majority of outdoor activity in infancy increased the risk of corneal pterygium by 20 times [9]. And there was a relative association between pterygium, sun exposure, and serum 25-hydroxyvitamin [10]. Furthermore, a study found that repeated exposure of the rabbit cornea to the same UVB irradiation evoked profound changes in corneal optics [11]. However, there was a lack of detailed information of UVB-induced damage on infancy cornea and relative protective measures.

Vitamin C, also known as ascorbic acid (AA), is initially identified as a key molecule in the prevention of scurvy and become more popular because of its antioxidant properties [12]. It is well known that the continuous stimulation of inflammation is the cause of many diseases [13]. Vitamin C could reduce oxidative stress production and inflammation to achieve protective effects. Researches showed that vitamin C could suppress UVB-induced cell death, apoptosis, ROS production, and the inflammatory response by downregulating tumor necrosis factor-*α* (TNF-*α*) expression [14, 15]. Moreover, UVB irradiation of vitamin-deficient animals resulted in severe corneal injury and -inflammatory response [16].

Nevertheless, a study showed that humans and other animals cannot synthesise vitamin C due to the lack of key enzymes that catalyze the last step of vitamin C biosynthesis [17]. Therefore, extra vitamin supplementation is a key source of vitamin C for the human body. Vitamin C acts as an electron donor and reduces the production of ROS such as superoxide radicals, hydroxyl radicals, and singlet oxygen and is widely used as an antioxidant to prevent many diseases. In addition to its role as an antioxidant, vitamin C is also a cofactor in collagen synthesis reactions. Moreover, study found UVB-induced changes in the corneal ultrastructure were most pronounced in animals fed an vitamin C-deficient diet. This suggests that ascorbic acid may play a vital role in protecting the corneal stroma from the harmful impacts of UVB [16, 18].

Therefore, the aim of this study was to explore the protective effects of vitamin C on cornea injury induced by UVB radiation in infant rats. The results would provide a new UVB prevention and protection perspective on infant cornea.

## 2. Materials and Methods

### 2.1. Animals

Thirty-six male Sprague-Dawley (SD) infant rats (2-3 weeks, 80-100 g) were obtained from the Animal Center of the Fourth Military Medical University (license No.2014270138S). The rats were raised and tested under the condition of free food and water intake and maintained room temperature of 23°C ± 3°C, humidity 45-65%, and 12 h light/12 h dark cycle. In this study, all the experimental protocols were approved by the ethical committee of the Animal Care and Experimental Committee of the Fourth Military Medical University. All experiments were in accordance with the Association for Research in Vision and Ophthalmology (ARVO) statements for the use of animals in ophthalmic research. Rats were euthanized using lethal sodium pentobarbital (Sigma, St Louis, MO, USA) at 7 d post-UVB irradiation.

### 2.2. UVB Irradiation Model

Twenty-four infant rats were anesthetized by intraperitoneal injection (IP) with 1% sodium pentobarbital (0.3 mL/100 g) and sumianxin II (composed of Jingsongling, ethylenediaminetetraacetic acid, dihydroetorphine hydrochloride, and haloperidol) (50 *μ*L each rat) (Jilin Shengda Animal Pharmaceutical Co., Ltd., Jilin, China). The UVB group infant rats, especially the eye tissues, were exposed to UVB irradiation (8 J/cm^2^, 15 min/d, 7 d) and the UVB+vitamin C group infant rats, especially the eye tissues, suffered from the same UVB exposure (8 J/cm^2^, 15 min/d, 7 d) added with vitamin C (40 mg/kg/d, 7 d, intraperitoneal administration). The control group was neither fed with vitamin C nor exposed to UVB irritation. The experiment lasted for 7 days. They were divided into three groups (CON group, UVB group, and UVB+VitC group), each group with twelve rats.

### 2.3. Slit Lamp Detection and OCT Detection

Slit lamp and OCT image detections were applied at 7 d post-UVB irradiation. Slit lamp detection was performed in a dark environment. Rats were anesthetized as previously described, and digital slit lamp (LS-5, Chongqing Shangbang Medical Equipment Co., Ltd.) detection was applied using direct and indirect methods as the operator manuals. The area of cornea desquamation and neovascularization was identified by Image-Pro Plus 6.0 software. The OCT detection procedure complied with the operator manual (Leica Bioptigen Envisu R2210, Leica Microsystems, Germany) using a 10 mm telecentric lens. The thickness of the cornea was calculated with an OCT Image Analysis Software.

### 2.4. HE Staining Examination

To visualize corneal histological changes, hematoxylin eosin (HE) staining of cornea eye sections was performed at 7 d post-UVB irradiation (*n* = 3). After the slit lamp examinations, rats were euthanized by intravenous lethal sodium pentobarbital. The eyeball was enucleated, and samples of the cornea were fixed in the solution containing 4% paraformaldehyde for 24 hours at 4°C. Six paraffin-embedded sections (thickness: 4 *μ*m) were cut along the transverse plane to enable discrimination of the nasal and temporal sides of each tissue slice. Then, the eye sections were prepared in the standard manner and stained with HE as previously described [19, 20].

### 2.5. Proinflammatory Cytokines and Oxidative Status in Serum

Infant rat blood was collected at 7 d postirradiation (*n* = 3). Proinflammatory cytokines, including interleukin-1 (IL-1), interleukin-6 (IL-6), and tumor necrosis factor-*α* (TNF-*α*) in the serum blood, were estimated using commercially available enzyme-linked immunosorbent assay (ELISA) kits (Sangong, China) according to the manufacturer's instructions. ROS-induced lipid peroxidation 4-hydroxynonenal (4-HNE) and malondialdehyde (MDA) were detected using ELISA kits (Sangong, China).

### 2.6. Immunofluorescence Staining

Immunofluorescence staining was performed according to the manufacturer's instructions at 7 d post-UVB irradiation (*n* = 3). Eye paraffin sections were deparaffinized in dimethylbenzene and dehydrated in gradient ethyl alcohol as previously described [20]. Then, the sections were washed with PBS (phosphate-buffered saline) (0.1 mM, pH = 7.2 ± 0.1) 3 times for 5 min. Antigen retrieval solution (9 mL 0.1 mmol/L citric acid + 41 mL 0.1 mmol/L sodium citrate + 450 mL ddH_2_0) was performed with a medium baking temperature for 10 min. Next, the sections were washed in PBS 3 times for 5 min. They were incubated with 10% goat serum for 2 hours, then the sections were incubated with anti-VEGF-*α* (GeneTex, GTX102643, USA) at 1 : 100 dilution at 4°C. The slides were washed with PBS and incubated with IgG (H+L) secondary antibody, Cy3 conjugate (Zhuangzhi, EK022, Xi'an, Shaanxi province, China) at 1 : 200 dilution for 1 hour. DAPI (100 ng/mL) was applied to stain the nuclear for 15 min. Images of slides were captured with a fluorescence microscope (BX53, Olympus, Japan).

### 2.7. Western Blotting

Infant rat corneal tissues (*n* = 4) were separated and homogenized on an ice plate with 100 *μ*L RIPA Lysis Buffer (Beyotime Biotechnology, China) added with proteinases/phosphatase inhibitor (1 : 100 ratio). Next, the lysates were centrifuged at 10,000 rpm at 4°C for 15 min to obtain the supernatant. The concentration of the protein was evaluated by a bicinchoninic acid (BCA) protein quantitation kit (Beyotime Biotechnology, China). An equal amount of protein was denatured by boiling with the loading sample buffer, and then 20 *μ*g protein from each sample was loaded, separated by sodium dodecyl sulfate-polyacrylamide gel electrophoresis using a gel (5% spacer gel, 12% separation gel). Next, the proteins were transferred onto a PVDF membrane at 100 V for 120 min. Membranes were incubated with 5% defatted milk solution for 2 hours at room temperature and then reacted with anti-VEGF-*α* antibody (1 : 1000, GTX102643, GeneTex), anti-SOD1 antibody (1 : 1000; #Ab13498, Abcam), anti-SOD2 antibody (1 : 1000; #Ab13533, Abcam), and anti-GAPDH antibody (1 : 1000; Zhuangzhi Bioscience Technology Company) at 4°C overnight. The membranes were then incubated with HRP-conjugated secondary antibody (1 : 10000; #EK020, Zhuangzhi Bioscience Technology Company) at room temperature for 1 hour, and then enhanced chemiluminescence (Thermo Scientific) was used for protein visualization. Intensity of immunoreactivity was quantified by densitometry using ImageJ software (NIH).

### 2.8. Statistical Analyses

Analysis of variance (ANOVA) followed by Bonferroni's post hoc analysis was performed to examine the statistical differences among all the groups unless otherwise specified; the values are presented as mean ± standard deviation (SD), with *p* ≤ 0.05 considered as statistically significant.

## 3. Results

### 3.1. UVB Irradiation Infant Rat Model

A UVB irradiation infant rat model was established by UVB irradiation (8 J/cm^2^, 15 min/d, 7 d). Twelve infant rats suffered UVB irradiation with vitamin C treatment (UVB+VitC group), and twelve infant rats suffered UVB irradiation without treatment (UVB group). 12 age-matched male infant rats served as the control (CON) group.

### 3.2. Effects of Vitamin C on Cornea Desquamation and Neovascularization

**Figure 1:**
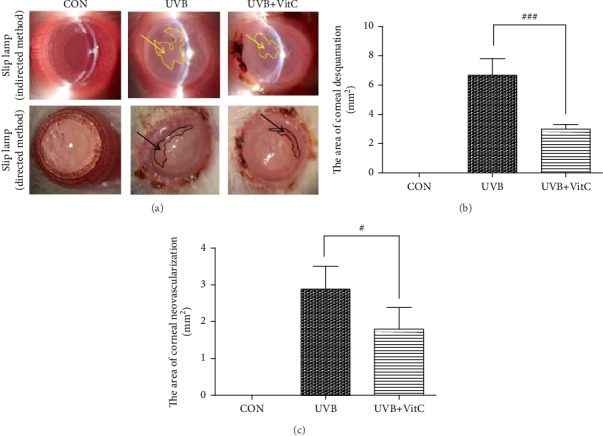
Vitamin C decreased the area of cornea desquamation and cornea neovascularization after UVB irradiation for 7 days. (a) The typical slit lamp picture with indirect method and direct method; (b) the area of cornea desquamation at 7 d post-UVB irradiation; (c) the area of cornea neovascularization 7 d post-UVB irradiation. All analyses were performed in duplicates. The data were expressed as mean ± standard deviation (SD), *n* = 3 rats per group. ^#^*p* < 0.05, UVB+VitC group vs. UVB group; ^###^*p* < 0.001, UVB+VitC group vs. UVB group.

To evaluate the damage severity of UVB irradiation on the cornea, the slit lamp detection was performed at 7 d post-UVB irradiation. Considering the corneal edema, fluorescein sodium staining was applied unsuccessfully. Therefore, we calculated the area of typical cornea desquamation at the half corneal field using the slit lamp with the indirect method. As shown in Figure 1, the results showed that the area of cornea desquamation in the UVB+VitC group was less than that in the UVB group (*p* < 0.05), and there existed no cornea desquamation in the normal infant rat cornea. The area of cornea neovascularization in the UVB+VitC group was less than that in the UVB group (*p* < 0.05). Moreover, there existed no neovascularization in the normal infant rat cornea.

### 3.3. Effects of Vitamin C on Cornea Structure

**Figure 2:**
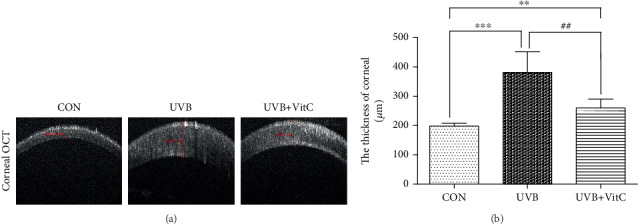
Vitamin C decreased the cornea edema after UVB irradiation for 7 days. (a) The typical cornea OCT picture at 7 d post-UVB irradiation. (b) The thickness of cornea at 7 d post-UVB irradiation. All analyses were performed in duplicates. The data were expressed as mean ± standard deviation (SD), *n* = 6 rats per group. ^∗∗^*p* < 0.01, UVB group and UVB+VitC group vs. CON group; ^∗∗∗^*p* < 0.001, UVB group and UVB+VitC group vs. CON group; ^##^*p* < 0.01, UVB+VitC group vs. UVB group.

**Figure 3:**
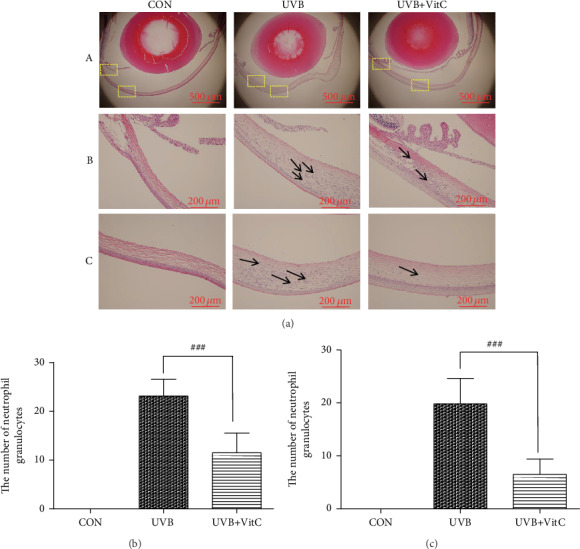
Vitamin C decreased the number of neutrophil granulocytes at the cornea after UVB irradiation for 7 days. (a) The typical HE staining picture; (b) the number of neutrophil granulocytes at the center of the cornea at 7 d post-UVB irradiation; (c) the number of neutrophil granulocyte at the limbic cornea at 7 d post-UVB irradiation. All analyses were performed in duplicates. The data are expressed as mean ± standard deviation (SD), *n* = 3 rats per group. ^###^*p* < 0.001, UVB+VitC group vs. UVB group.

To evaluate the corneal function post-UVB irradiation treated or untreated with vitamin C, OCT detection and HE staining were performed. As shown in Figure 2, the thickness of the central cornea in both the UVB group (381.4 ± 70.8 *μ*m) and the UVB+VitC group (260.2 ± 30.7 *μ*m) was increased compared with that in the CON group (198.0 ± 9.9 *μ*m) at 7 d post-UVB irradiation (all *p* < 0.05). Furthermore, the cornea in the UVB+VitC group was less edematous than that in the UVB group (*p* < 0.05). Moreover, HE staining results showed that the epithelial tissues of the CON group were arranged neatly, and the collagen fibers in the stroma were arranged parallel, regular, and compact. Epithelial edema, structural disorder, and neovascularization were observed in the UVB group. Interestingly, the corneal structure of the UVB+VitC group was basically intact; epithelial edema was alleviated (Figure 3(a)).

### 3.4. Effects of Vitamin C on Cornea Acute Inflammation

To evaluate corneal inflammation post-UVB irradiation treated or untreated with vitamin C, HE staining and serum inflammatory factors detection were performed. HE staining showed the cornea edema, which was consistent with OCT findings (Figure 3(a)). Meantime, a great number of neutrophil granulocytes were found from the limbic cornea to the central cornea after UVB irradiation for 7 days. As we could see, the number of neutrophil granulocytes at both the limbic cornea and the central cornea in the UVB group (23.2 ± 3.4/high power vision; 19.8 ± 4.8/high power vision) was more than those in the UVB+VitC group (11.5 ± 4.1/high power vision; 6.5 ± 2.9/high power vision) (all *p* < 0.05), while there existed no neutrophil granulocyte at the cornea in the CON group (Figures 3(b) and 3(c)).

**Figure 4:**
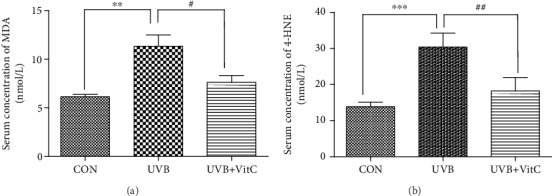
Vitamin C decreased the serum level of MDA and 4-HNE after UVB irradiation for 7 days. (a) The serum level of MDA at 7 d post-UVB irradiation; (b) the serum level of 4-HNE at 7 d post-UVB irradiation. All analyses were performed in duplicates. The data were expressed as mean ± standard deviation (SD), *n* = 3 rats per group. ^∗∗^*p* < 0.01, UVB group and UVB+VitC group vs. CON group; ^∗∗∗^*p* < 0.001, UVB group and UVB+VitC group vs. CON group; ^#^*p* < 0.05, UVB+VitC group vs. UVB group; ^##^*p* < 0.01, UVB+VitC group vs. UVB group.

**Figure 5:**
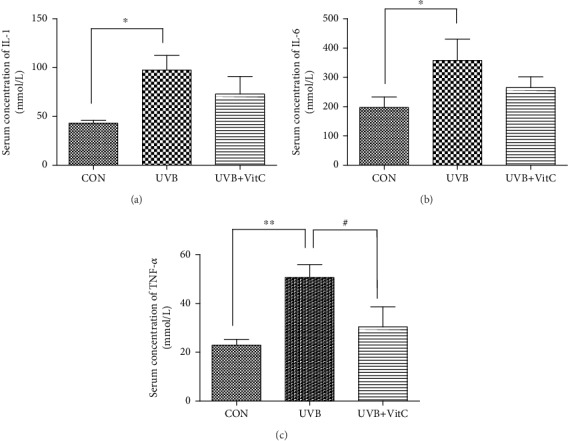
Vitamin C decreased serum level of IL-1, IL-6, and TNF-*α* after UVB irradiation for 7 days. (a) The serum level of IL-1 at 7 d post-UVB irradiation; (b) the serum level of IL-6 at 7 d post-UVB irradiation; (c) the serum level of TNF-*α* at 7 d post-UVB irradiation. All analyses were performed in duplicates. The data were expressed as mean ± standard deviation (SD), *n* = 3 rats per group. ^∗^*p* < 0.05, UVB group and UVB+VitC group vs. CON group; ^∗∗^*p* < 0.01, UVB group and UVB+VitC group vs. CON group; ^#^*p* < 0.05, UVB+VitC group vs. UVB group.

Moreover, ROS-induced lipid peroxidation 4-hydroxynonenal (4-HNE) and malondialdehyde (MDA) in the serum was increased after UVB irradiation for 7 days. And the serum concentrations of 4-HNE and MDA in the UVB+VitC group were significantly decreased compared with those in the UVB group (all *p* < 0.05) (Figures 4(a) and 4(b)). Additionally, serum inflammatory factors IL-1, IL-6, and TNF-*α* were increased in varying degrees after UVB irradiation for 7 days. The serum level of IL-1 and TNF-*α* in the UVB+VitC group were significantly reduced compared with those in the UVB group (all *p* < 0.05) (Figures 5(a)–5(c)).

### 3.5. Effects of Vitamin C on the Expression of Corneal VEGF-*α* and SOD

**Figure 6:**
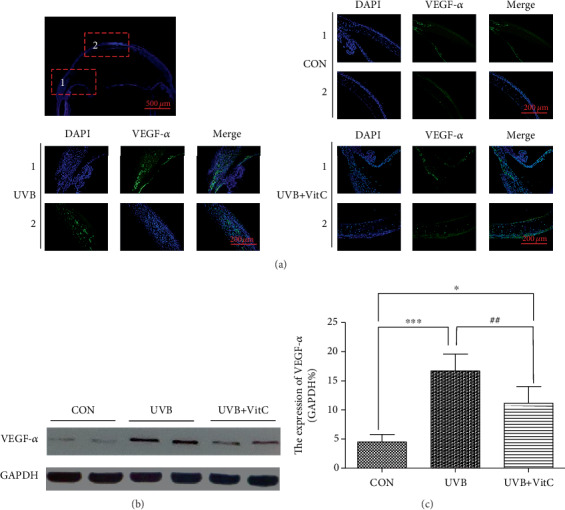
Vitamin C decreased the expression of VEGF-*α* after UVB irradiation for 7 days. (a) The typical immunofluorescence staining picture of VEGF-*α* at 7 d post-UVB irradiation; (b) the typical western blot picture of VEGF-*α* at 7 d post-UVB irradiation; (c) the expression of VEGF-*α* detected by western blot detection at 7 d post-UVB irradiation. All analyses were performed in duplicates. The data were expressed as mean ± standard deviation (SD), *n* = 3 rats per group. ^∗^*p* < 0.05, UVB group and UVB+VitC group vs. CON group; ^∗∗∗^*p* < 0.001, UVB group and UVB+VitC group vs. CON group; ^##^*p* < 0.01, UVB+VitC group vs. UVB group.

To evaluate the expression of corneal VEGF-*α* and SOD in rats treated or untreated with vitamin C, immunofluorescence staining, western blot, and ELISA detections were applied. As shown in Figure 6(a), VEGF-*α* expressed mainly in the limbus and corneal endothelium at normal conditions. And when exposed to UVB irradiation for 7 days, VEGF-*α* expressed at the whole cornea, including the corneal epithelial layer, stromal layer, and corneal endothelium. Furthermore, the expression of VEGF-*α* in the UVB group and UVB+VitC group at 7 d post-UVB irradiation was dramatically increased compared with the CON group (all *p* < 0.05), while the expression of VEGF-*α* in the UVB+VitC group was less than that in the UVB group (*p* < 0.05) as confirmed by western blot (Figures 6(b) and 6(c)).

**Figure 7:**
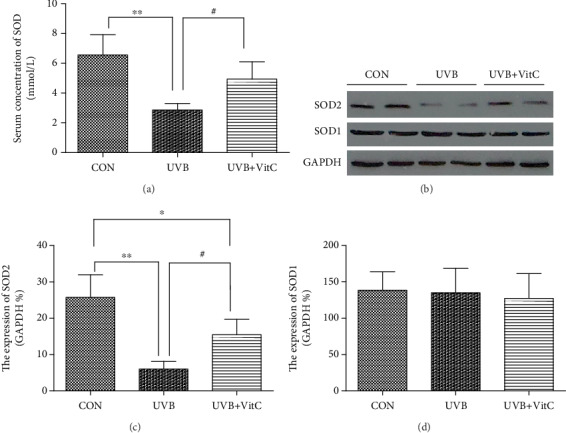
Vitamin C increased the expression of SOD (SOD2) after UVB irradiation for 7 days. (a) The serum concentration of SOD detected by ELISA at 7 d post-UVB irradiation; (b) the typical western blot picture of SOD1 and SOD2 at 7 d post-UVB irradiation; (c) the expression of SOD2 detected by western blot at 7 d post-UVB irradiation; (d) the expression of SOD1 detected by western blot at 7 d post-UVB irradiation. All analyses were performed in duplicates. The data were expressed as mean ± standard deviation (SD), *n* = 3 or 4 rats per group. ^∗^*p* < 0.05, UVB group and UVB+VitC group vs. CON group; ^∗∗^*p* < 0.01, UVB group and UVB+VitC group vs. CON group; ^∗∗∗^*p* < 0.001, UVB group and UVB+VitC group vs. CON group; ^#^*p* < 0.05, UVB+VitC group vs. UVB group; ^##^*p* < 0.01, UVB+VitC group vs. UVB group.

ELISA results found that the serum level of SOD in the UVB group at 7 d post-UVB irradiation was dramatically decreased compared with that in the CON group (*p* < 0.05), while the UVB+VitC group showed a higher SOD level compared with the UVB group (*p* < 0.05) (Figure 7(a)). Meanwhile, western blot results showed that the expression of SOD2 in corneal tissue homogenization in the UVB group was significantly decreased compared with that in the CON group (*p* < 0.05), while the UVB+VitC group showed a higher SOD2 expression compared with the UVB group (*p* < 0.05) (Figures 7(b) and 7(c)). Moreover, there was no significant difference in the SOD1 expression among the UVB group, UVB+VitC group, and CON group at 7 d post-UVB irradiation (all *p* > 0.05) (Figure 7(d)).

## 4. Discussion

Our results showed that the corneas of infant rats were vulnerable to ultraviolet radiation. UVB exposure led to corneal epithelial damage, cell edema, structural disorders of the stroma, and infiltration of inflammatory cells. Moreover, vitamin C could reduce the corneal injury area, improve corneal tissue structure, also decrease systemic inflammation, and increase antioxidant levels.

Research has shown that UV radiation in sunlight resulted in certain damages to the human body [21]. Eyes were one of the organs sensitive to UV exposure, and a study showed that a series of eye diseases were associated with acute or chronic UV damage [22]. Studies had found that the mechanism of corneal injury induced by UV radiation is mainly caused by photochemical reaction rather than thermal reaction [23]. The key role of UV radiation reaction is the process of converting physical energy into biochemical reaction through target molecules; after UV irradiation, hydroxyl radicals are produced in photochemistry, and free radicals trigger inflammation, leading to tissue damage [22]. A study showed that in humans, a cornea from a 24-year-old could absorb over 90% of UVB and 45% of UVA [6].

Several studies had shown that excessive production of ROS and antioxidant mechanism imbalance were the main reasons that caused inflammation after acute UVB irritation and the degradation process associated with chronic UVB damage [24]. UVB exposure increased ROS production and decreased antioxidant levels in tissues, and this disruption of intracellular redox homeostasis could promote cell death [25]. Moreover, UVB irradiation activated oxidative stress-related transcription factors, such as nuclear factor-*κ*B (NF-*κ*B), and induced the production of many cytokines [26]. Our results showed that inflammatory cytokines, including TNF-*α*, IL-1, and IL-6, were obviously increased in the blood after UVB irradiation. Furthermore, the expression of SOD (SOD2, located at the mitochondrion), the antioxidative macromolecule, was dramatically decreased after UVB irradiation.

UVB-induced acute damage in the cornea could be heavily attributed to the upregulation of proinflammatory cytokines, which was confirmed by both animal experiments and cell experiments [27, 28]. Recently, studies showed that UVB exposure could induce DNA damage, limbal epithelial cell differentiation, and secretion of proinflammatory cytokines in cultured limbal epithelial cells [29]. In this study, we identified the corneal complications induced by UVB irradiation, mainly including corneal edema, and neovascularization.

Many studies have reported that natural antioxidants are efficacious in preventing and curing the UVB-induced corneal pathology due to their particular interactions and synergism. Studies showed corneal injury induced by UVB in adult rabbits and observed the protective effects of vitamin C [15, 30]. Meantime, a vitamin C-deficient diet showed that chronic UVB irradiation leaded to increasing corneal thickness [16, 18]. These evidences suggests that vitamin C could play an essential role in protecting the corneal injury from UVB. However, there were no reports of corneal damage and protective measures of UVB radiation in infant rats. Our results showed that UVB significantly damaged 3-week-old rats' cornea, leading to severe corneal edema and structural disorders, as well as inflammatory reactions. Interestingly, vitamin C could downregulate proinflammatory cytokines and prevent the activation of neutrophil granulocytes, which reduced inflammatory reactions.

Relative studies showed that the expression of VEGF-*α*, a neovascularization factor, in the UVB-irradiated cornea was dramatically increased, and the UVB-irradiated cornea was vascularized and nontransparent [31]. It was known that the excessive generation of VEGF-*α* was accompanied with histiocyte hypoxia, which was associated with further vascular leakage and cornea edema. Moreover, vitamin C could directly improve the retina hypoxia condition, and the potential mechanisms could be related to the selective elimination of strong free radicals and inhibition of inflammatory factors and activationof cell survival signals. In our study, similar results were found in the UVB group, while vitamin C could alleviate those inflammatory cytokines. The results of this study showed that extra vitamin C supplementation could ameliorate corneal injury caused by UVB, reduce corneal edema, improve corneal structure, increase antioxidant productions, and alleviate inflammation.

## 5. Conclusion

In summary, acute UVB irradiation could lead to a variety of pathological changes in the cornea, including damages of the epithelium, stromal cell, and endothelium. Moreover, vitamin C, as a natural antioxidant, could protect corneal structure integrity via alleviating corneal edema, improving corneal inflammatory reaction, and regulating VEGF-*α* expression. Taken together, the study provided an effective support that vitamin C may be a good candidate for protective agents when exposed to heavy sunlight.

## Data Availability

The data sets used and analyzed in the present study are available from the corresponding author on reasonable request.

## Abbreviations

AA: Ascorbic acid
ARVO: Association for Research in Vision and Ophthalmology
IL-1: Interleukin-1
IL-6: Interleukin-6
NF-*κ*B: Nuclear factor-*κ*B
OS: Oxidative stress
ROS: Reactive oxygen species
SOD: Superoxide dismutase
SD: Sprague-Dawley
TNF-*α*: Tumor necrosis factor-*α*
UVA: Ultraviolet A
UVB: Ultraviolet B
UVC: Ultraviolet C
VEGF-*α*: Vascular endothelial growth factor-*α*.
